# Change in bone mineral density after cemented and uncemented knee arthroplasty with an asymmetrical tibial component: secondary analysis of a randomized study using dual-energy X-ray absorptiometry

**DOI:** 10.2340/17453674.2026.45944

**Published:** 2026-06-04

**Authors:** Müjgan Yilmaz ALTUN, Gunnar FLIVIK, Thomas LIND, Anders ODGAARD, Michael Mørk PETERSEN

**Affiliations:** 1 Department of Orthopedic Surgery, University Hospital of Copenhagen, Rigshospitalet, Copenhagen; 2 Department of Orthopedic Surgery, University Hospital of Copenhagen, Herlev-Gentofte Hospital, Hellerup; 3 Department of Orthopedics, Skane University Hospital, Clinical Sciences, Lund University, Lund; 4 Department of Clinical Medicine, Faculty of Health and Medical Sciences, University of Copenhagen, Denmark

## Abstract

**Background and purpose:**

Total knee arthroplasties (TKA) affect the mechanical loading of the knee joint and may be associated with changes in bone mineral density (BMD). We aimed to evaluate adaptive periprosthetic BMD after cemented and uncemented TKA.

**Methods:**

This is a secondary report of an earlier published RCT. Patients receiving cemented (n = 31) or uncemented (n = 32) TKA were included in a randomized controlled trial (RCT) with a 1:1 allocation. BMD was measured using Dual-energy X-ray Absorptiometry (DEXA) at 1 week and 3, 6, 12, and 24 months postoperatively in 3 regions of interest (ROI) in the femur and tibia. Changes in BMD were assessed using a paired t-test, and between-groups differences using an unpaired t-test. Time-related changes were analyzed using ANOVA. The study was registered at clinicaltrials.gov (NCT03563131) before enrolment.

**Results:**

Femoral components: Over 2 years, BMD in ROI I decreased by 33% in the uncemented group and 21% in the cemented group, with a between-group difference of 12.2 percentage points (95% confidence interval [CI] 5.3–19.1; significant). In ROI II, the decrease was 19% vs 13%, with a between-group difference of 6.1 percentage points (CI –1.2 to 13.5; not significant). In ROI III, decreases were 6% vs 7%, with a between-group difference of –0.6 percentage points (CI –4.3 to 3.3; not significant). Tibial components: Changes were small (-4.7 to 3.3%), with significant decreases only in ROI I in the cemented group over 24 months. No significant between-group differences were observed.

**Conclusion:**

The periprosthetic BMD after TKA decreased both around cemented and uncemented components, particularly in ROI I after using an uncemented femoral component, whereas the decrease under the tibial components was small and of uncertain clinical significans.

Symptomatic advanced osteoarthritis (OA) is successfully treated with total knee arthroplasty (TKA), with implant survival rates around 95% at 10 years [1]. However, TKA affects the mechanical loading of the knee joint, which is associated with changes in bone mineral density (BMD) [2,3]. Periprosthetic BMD changes may be clinically important, as reduced BMD is associated with a decrease in the breaking strength of the bone [4].

Following TKA, a decrease in BMD is expected due to the surgical trauma and postoperative immobilization [5,6]. Subsequently, stress-shielding may further contribute to BMD loss [5,6]. The femoral component reduces patellofemoral load to the anterior distal femur, leading to stress-shielding and osteopenia in this region [7,8]. Previous studies indicate that the anterior distal femur exhibits the majority of BMD decrease (up to 44%) [8,9], theoretically increasing the risk of periprosthetic fractures or loosening of the component [10].

The decrease in BMD around the tibial component is primary located in the medial plateau and may be caused by stress-shielding [11,12], with a reported decrease of up to 41% [2,9]. However, some studies have demonstrated unchanged or only mild decrease in the proximal tibia BMD at 1 year postoperatively, regardless of fixation mode [13,14].

To our knowledge, comparative studies evaluating adaptive bone remodeling after TKA with respect to implant fixation mode (cemented vs uncemented) and component design remains limited.

This is a secondary report of a randomized controlled trial (RCT) [15]. The aim of our study was to compare adaptive bone remodeling following TKA using an uncemented trabecular metal (TM) component vs a cemented Persona TKA components assessed by Dual-energy X-ray Absorptiometry (DEXA).

## Methods

### Study design and patients

Data was prospectively collected, and randomization was performed in blocks of 10 with 1:1 allocation to either fully cemented or uncemented fixation of TKA components. Allocation was performed intraoperatively by a nurse not involved in the study, using sealed envelopes after sedation and before skin incision. Surgeons were not blinded due to differences in prosthesis design and fixation, whereas patients remained blinded throughout the 2-year follow-up.

Inclusion criteria were TKA due to OA performed at Gentofte Hospital Department of Orthopedic Surgery between September 2018 and October 2019, age between 40 and 70 years, and cognitively fit to understand and sign an informed consent. Patients with conditions that could affect bone metabolism, or who were unable to provide informed consent, were excluded.

### Surgical technique

All procedures were performed under either spinal or general anesthesia. A midline skin incision was made, followed by a medial parapatellar arthrotomy to expose the knee joint. The distal femur and proximal tibia were resected using alignment guides from Zimmer Biomet (Persona system) to ensure restoration of the mechanical axis and accurate component positioning. All patients received a cruciate-retaining polyethylene insert and a cemented all-polyethylene patellar component. In the cemented group, fixation was achieved using a pressurized application of cement to both the prepared bone surfaces and the implant interfaces.

### DEXA and follow-up

DEXA measurements of the distal femur and proximal tibia on the surgical limb, as well as both ankles, were performed at 1 week, 3 months, 6 months, 12 months, and 24 months postoperatively. All measurements were performed at the Department of Orthopedic Surgery at Rigshospitalet, Copenhagen, Denmark, by an experienced research nurse.

A double examination was performed at the 12-month follow-up. Subsequent to the first examination, the patient was requested to stand up, walk around, and again placed in position and rescanned.

To obtain measurements of the proximal tibia and both ankles, the patient was placed in a standardized supine position, the ankles fixed with a block, and weights were placed over the anterior crus to minimize movements. A small internal rotation was applied to minimize overlayer of the proximal tibia and fibula.

To obtain measurements of the distal femur, the patient was placed on the side of the surgical limb, with a small flexion in the knee and with weights over the ankle.

All measurements were performed with a Norland XR-46 bone densitometer (Norland Corp, Fort Atkinson, WI, USA), with a scan speed of 45 mm/s and a pixel size of 0.5 x 0.5 mm.

Prior to DEXA measurements, a daily calibration was performed for quality control. For analyses, customized software was used to regulate the threshold for metal exclusion, allowing measurements of the bone adjacent to the component.

**Figure 1:**
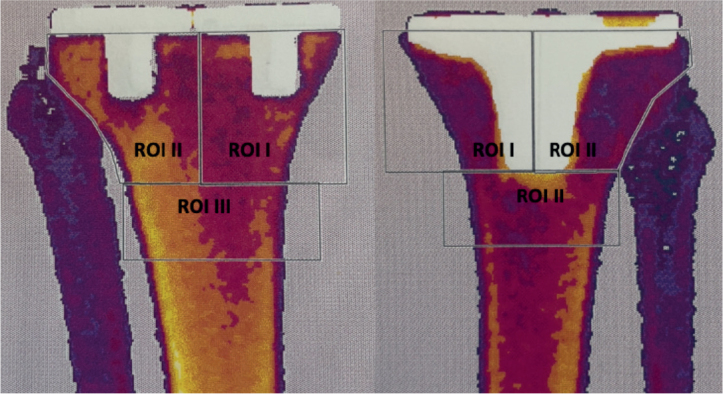
ROI in cemented (right) and uncemented (left) tibial components.

The DEXA images were analyzed in the region of interest (ROI). To enable and distinguish the analyses of the BMD in the bone related to the tibial and femoral components, the images were divided into 3 different ROIs. The tibial component was divided into 2 equal halves, and a 4 cm vertical line was drawn from the proximal to the distal part of the component (Figure 1). The medial half was named ROI I, and the lateral part ROI II. A vertical line of 2 cm drawn from the distal part of ROI I and II, including the whole bone segment, formed the last ROI III (Figure 1).

**Figure 2:**
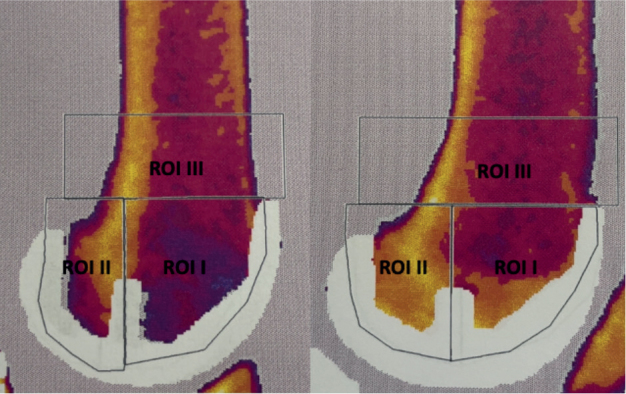
ROI in cemented (right) and uncemented (left) femoral components.

Similarly, the femoral component was divided into an anterior and posterior part. A vertical line through the pegs (2 small pins in the distal and middle of the component) was drawn and once meeting a horizontal line from the apex of the femoral component, the top of ROI I and II was formed (Figure 2). The anterior part was named ROI I, and the posterior part ROI II. A proximal part, ROI III, was located 2 cm proximal to ROI I and II and included the whole bone segment (Figure 2). All BMD measurements were measured in g/cm^2^.

### Sample size considerations and study hypothesis

The primary outcome of this study was the local changes in BMD of the various ROIs at 2 years of follow-up, and our hypothesis was that the uncemented TM TKA would have less loss of BMD compared with the cemented version of the implant.

The sample size calculations were done using data from a study at the time of study protocol writing in a recently published 1-year follow-up DEXA study [16]. Using 1-year follow-up data was justified because the decrease in BMD seen beyond the first postoperative year was very limited in most previously published studies [5,7,17–19].

A difference of 8% between groups was assessed to be clinically meaningful and to provide a sample size with a high enough statistical power when comparing 2 fixation techniques. Using a type I error of 5%, 90% statistical power, minimal relevant difference of 8%, and standard deviation of 8.4% we calculated the sample size.

Although the calculation calls for 25 patients in each group, it was done with 60 patients—30 in each group—due to expected dropouts. Early dropouts were observed in our trial; as a result, 66 people were randomly assigned, leaving 63 patients for further follow-up.

### Statistics

The mean coefficient of variation (CV [%] = standard deviation [SD]/mean x 100) expresses the precision error of the BMD analyses in the different ROI of the proximal tibia and the distal femur.

The BMD data was checked for distribution, and statistical tests were applied accordingly. An unpaired t-test was used to evaluate differences in BMD between the cemented and uncemented groups of femoral and tibial components after 24 months. A paired t-test was used to evaluate the change from the first postoperative measurement to the measurement at 24 months.

Time-related changes in groups were evaluated with ANOVA. Statistical significance was set to P < 0.05, and 95% confidence intervals were reported as CI. Analyses are valid assuming that data was Missing Completely At Random [20]. Statistical analyses were performed in RStudio (Version 1.2.1335 © 2009–2019; R Foundation for Statistical Computing, Vienna, Austria).

### Ethics, data sharing plan, funding, use of AI, and disclosures

Approval from the local Ethics Committee (case no. H-16035883) and Danish Data Protection Agency (case no. 2012-58-0004, RH-2017-36 and I-Suite nr: 05264) were obtained.

All patients were informed orally and in writing by the principal investigator and before inclusion informed consent was obtained by following the Helsinki Declaration.

Before inclusion, the randomized controlled study was registered at clinicaltrial.gov (protocol ID: PERSONA-RH-18, clinicaltrial.gov ID: NCT03563131).

The datasets used and/or analyzed during the current study are available from the corresponding author upon reasonable request.

MYA and TL have nothing to disclose. GF reports institutional grants from Zimmer-Biomet, outside the submitted work. Furthermore, GF reports institutional grants outside the submitted work from Stryker, Depuy Synthes, JRI Ltd, Materialize, and Ortoma. AO reports institutional grants from Zimmer-Biomet, outside the submitted work. MMP reports institutional grants from Zimmer Biomet, during the conduct of the study; grants from Ethicon UK, and grants from Zimmer Biomet, outside the submitted work. Grants from Zimmer Biomet were given to Rigshospitalet during the conduct of the study. Complete disclosure of interest forms according to ICMJE are available on the article page, doi: 10.2340/17453674.2026.45944

## Results

**Figure 3:**
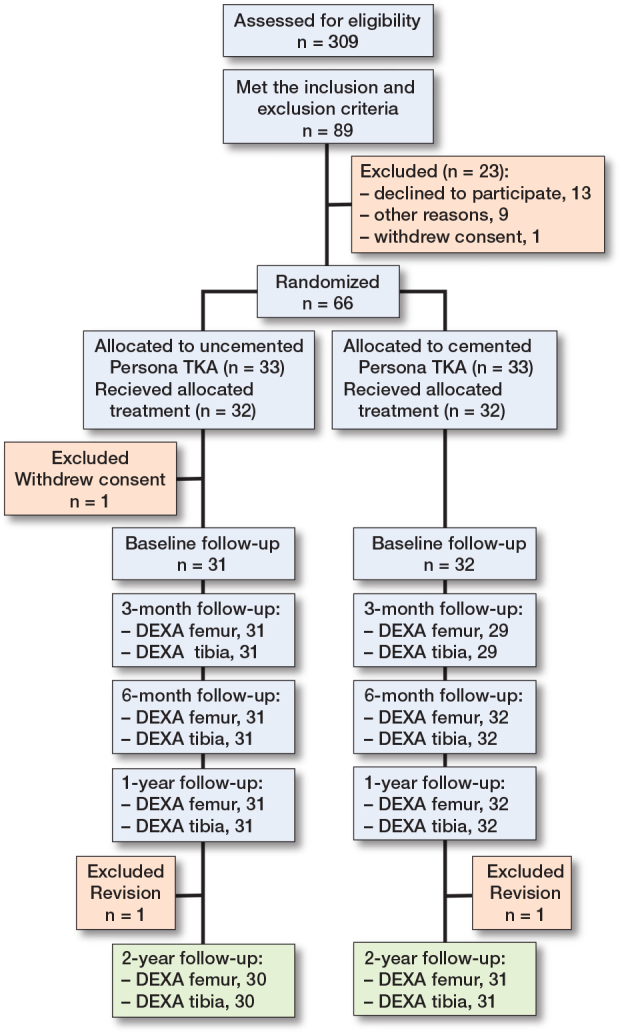
Enrolment.

66 patients were included in the study; however, 2 patients did not receive the allocated treatment as they did not meet the criteria for cruciate-retaining TKA and were subsequently excluded. 1 patient from the cemented group withdrew consent after the allocation and was excluded. A total of 63 patients were included for further follow-up. 32 patients received uncemented femoral and tibial components featuring a TM surface whereas 31 patients received a fully cemented prosthesis (Figure 3, Table 1).

**Table 1:** Demography of the cohort

	Total (n = 63)	Cemented (n = 32)	Uncemented (n = 31)
Mean age at surgery (range)	62 (50–71)	61 (50–70)	64 (51–71)
Body mass index (SD)	30.2 (5.1)	30.5 (5.6)	29.8 (4.5)
range	21.6–46.1	21.6–46.1	23.0–40.4
Sex			
Female	41	22	19
Male	22	10	12
Smoking			
Never	31	14	17
Current	8	3	5
Former	24	15	9
ASA grade			
1	15	9	6
2	46	22	24
3	2	1	1
Anesthesia type			
General	27	15	12
Spinal	36	17	19
Surgery side			
Left	32	18	14
Right	31	14	17
Prosthesis components			
Femoral size			
4	2	1	1
5	2	2	0
6	8	5	3
7	12	6	6
8	18	8	10
9	11	5	6
10	6	2	4
11	2	1	1
12	2	2	0
Standard	48	23	25
Narrow	15	9	6
Tibial size			
C	1	0	1
D	18	10	8
E	15	10	5
F	17	6	11
G	7	2	5
H	5	4	1
Insert thickness (range), mm	12 (10–18)	12 (10–18)	11 (10–14)
Patella diameter (range), mm	34 (29–41)	34 (29–41)	35 (29–41)

ASA = American Society of Anesthesiologists Physical Status classification

### Precision error measurements

*Femur:* Precision error for ROI I (anterior) was 1.86% (CI 1.45–2.28), ROI II (posterior) was 1.6% (CI 1.23–1.97), and ROI III (proximal) was 1.39% (CI 1.06–1.72).

*Tibia:* Precision error for ROI I (medial) was 2.12% (CI 1.67–2.56), ROI II (lateral) was 2.5% (CI 1.84–3.17), and ROI III (distal) was 2.13% (CI 1.50–2.77).

### Change in BMD

#### Femoral component

**Figure 4:**
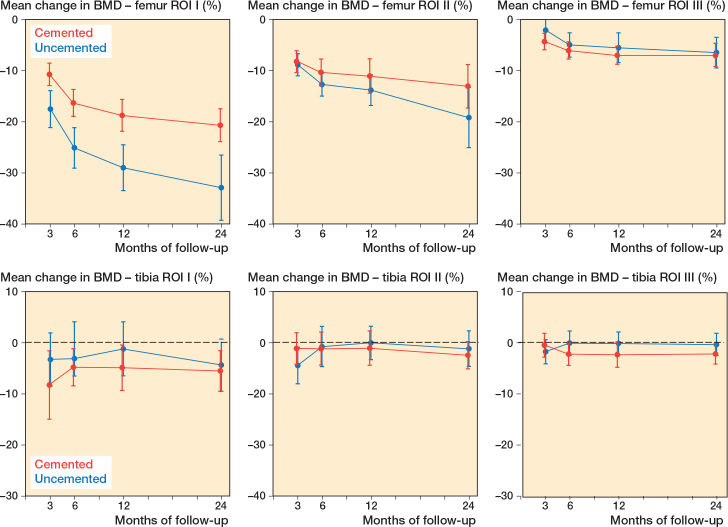
Changes in femoral BMD (upper panels) and tibial BMD lower panels for ROIs I–III; whiskers indicate 95% CI.

The femoral BMD in ROI I decreased by 33% (CI 27–39) in the uncemented group and by 21% (CI 17–24) in the cemented group over 2 years; the between-group difference was 12.2 percentage points (CI 5.3–19.1). We found statistically significant differences between the fixation types throughout all follow-up measurements with less decrease in BMD in the cemented group (Table 2, Figure 4).

**Table 2:** Percentwise changes in BMD from baseline examination presented as mean and (95% confidence interval)

ROI Time (months)	Cemented	Uncemented	Between-group difference
Femur ROI I			
3	–10.7 (–12.9 to –8.5)	–17.5 (–21.1 to –13.9)	6.8 (3.0 to 10.5)
6	–16.3 (–18.9 to –13.7)	–25.1 (–29.1 to –21.1)	8.8 (4.4 to 13.2)
12	–18.7 (–21.9 to –15.6)	–29.0 (–33.5 to –24.5)	10.2 (5.4 to 15.1)
24	–20.7 (–23.9 to –17.4)	–32.9 (–39.3 to –26.5)	12.2 (5.3 to 19.1)
Femur ROI II			
3	–8.2 (–10.4 to –6.1)	–8.9 (–11.0 to –6.7)	0.6 (–2.4 to 3.7)
6	–10.7 (–13.0 to –7.7)	–12.7 (–15.0 to –10.4)	2.4 (–1.1 to 5.8)
12	–11.1 (–14.4 to –7.8)	–13.8 (–16.8 to –10.9)	2.7 (–1.7 to 7.1)
24	–13.1 (–17.3 to –8.8)	–19.2 (–25.1 to –13.3)	6.1 (–1.2 to 13.5)
Femur ROI III			
3	–4.3 (–5.9 to –2.7)	–2.1 (–4.6 to 0.4)	–2.2 (–5.4 to 1.0)
6	–6.1 (–7.7 to –4.4)	–5.0 (–7.3 to –2.6)	–1.1 (–3.9 to 1.7)
12	–7.0 (–8.8 to –5.3)	–5.5 (–8.4 to –2.6)	–1.5 (–4.9 to 1.9)
24	–7.0 (–9.2 to –4.8)	–6.5 (–9.5 to –3.5)	–0.6 (–4.3 to 3.3)
Tibia ROI I			
3	–8.2 (–14.9 to –1.6)	–3.3 (–8.5 to 1.9)	–4.9 (–12.3 to 2.4)
6	–4.8 (–8.4 to –1.1)	–3.1 (–6.5 to 4.1)	–1.7 (–7.2 to 3.9)
12	–4.9 (–9.3 to –0.4)	–1.2 (–6.5 to 4.1)	–3.7 (–9.5 to 2.2)
24	–5.5 (–9.4 to –1.6)	–4.4 (–9.5 to 0.7)	–1.1 (–7.3 to 5.1)
Tibia ROI II			
3	–1.1 (–4.2 to 1.9)	–4.4 (–8.0 to –0.8)	3.3 (–1.5 to 8.1)
6	–1.2 (–4.4 to 2.0)	–0.8 (–4.7 to 3.2)	–0.4 (–5.9 to 5.0)
12	–1.1 (–4.4 to 2.3)	–0.1 (–3.3 to 3.2)	–1.0 (–6.3 to 4.3)
24	–2.5 (–5.1 to 0.2)	–1.2 (–4.7 to 2.3)	–1.3 (–5.9 to 3.4)
Tibia ROI III			
3	–0.5 (–2.8 to 1.8)	–1.8 (–4.1 to 0.6)	1.3 (–2.1 to 4.6)
6	–2.3 (–4.4 to –0.1)	–0.1 (–2.5 to 2.3)	–2.2 (–5.3 to 1.0)
12	–2.4 (–4.8 to 0.1)	–0.1 (–2.4 to 2.1)	–2.2 (–5.4 to 1.0)
24	–2.2 (–4.2 to –0.2)	–0.4 (–2.5 to 1.8)	–1.8 (–4.7 to 1.1)

The femoral BMD in ROI II decreased by 19% (CI 13–25) in the uncemented group and by 13% (CI 9–17) in the cemented group over 2 years; the between-group difference was 6.1 percentage points (CI –1.2 to 13.5), with no statistically significant difference between the fixation types in ROI II (Table 2, Figure 4).

The femoral BMD in ROI III decreased by 6% (CI 3–9) in the uncemented group and by 7% (CI 5–9) in the cemented group over 2 years; the between-group difference was –0.6 percentage points (CI –4.3 to 3.3), with no statistically significant difference between the fixation types in ROI III (Table 2, Figure 4).

#### Tibial component

The changes in BMD below the tibia component were limited, and a decrease after 2 years of follow-up between 0.4% and 5.5% was seen. Statistically significant changes were found only in ROI I (uncemented group) and ROI II (cemented group), with a decrease in BMD from baseline to the 2-year follow-up of respectively 6% (CI 2–9) and 2% (CI 0–4).

We found no statistically significant difference in the changes between the fixation types in the 3 ROIs (Table 2, Figure 4).

#### Ankles

No significant change in BMD was found when comparing the ankle of the surgical limb with the contralateral side as well as the same site with baseline and 24-month follow-up.

## Discussion

We aimed to compare change in BMD between cemented and uncemented TKA. The bone area with the greatest change in BMD was femoral ROI I, with a decreased of 33% in the uncemented group and 21% in the cemented group and a between-group difference of 12.2 percentage points. ROI I is the anterior part of the femoral bone behind the anterior flange. This area is of notable clinical interest as previous studies have shown the largest decrease in BMD subsequent to TKA in this anatomical location, which can increase the risk of periprosthetic fractures that require revision surgery [10,21]. Several factors can lead to revision surgery, of which aseptic loosening is the leading cause [22,23].

The risk of aseptic loosening and periprosthetic fractures in the distal femur could be associated with the large decrease in BMD observed in relation to the femoral component [22].

We observed the largest decrease in BMD in the anterior part of the femoral component within the first 3 months postoperatively with a tendency of stabilization throughout the follow-up period but without reaching a steady state. Several factors can influence the decrease in BMD, of which surgical trauma is considered to cause most of the initial decrease, whereas the subsequent decrease could be caused by immobilization or stress-shielding [7,8]. Our results are in concordance with previous studies that have reported a mean decrease of up to 44% under the anterior flange of the distal femur after 2 years [8,17].

In our study, the largest decrease in BMD related to the tibial component was found in ROI I, the medial part of the component with a decrease of 5.5% in the cemented group and 4.4% in the uncemented group. We did not find any significant difference between the fixation types related to the tibial component. Our findings are smaller than those reported in previous studies where a mean medial decrease of up to 41% has been reported [2,9,12,24].

We did not find any difference in the BMD when comparing the ankle on the surgical limb with the contralateral ankle, which indicates that the decrease in BMD observed in the bone related to the femoral and tibial components is not likely to be caused by immobilization as it then would be expected to observe a similar decrease in the ankle region. Instead, the decrease in BMD can be assumed to be caused by local adaptation and bone remodeling.

### Limitations

The postoperative measurements were performed with a mean of 7 days after surgery and we do not know if any significant bone remodeling takes place within this week.

An important consideration when interpreting results related to a decrease in BMD following TKA is that local changes caused by the disease might have increased the preoperative BMD [25]. Therefore, the decrease seen postoperatively could be the result of the BMD returning to “normal” condition rather than an actual low BMD caused by the surgery.

### Conclusion

The periprosthetic BMD after TKA decreased both around cemented and uncemented components, particularly in ROI I after using an uncemented femoral component, whereas the decrease under the tibial component was small and of uncertain clinical significans.

*In perspective,* a clinically relevant difference in bone remodeling at 24 months, favoring cemented fixation, could potentially reduce the risk of implant-related complications such as periprosthetic fractures on the femoral side.

## Notes

MYA: conducted data analysis, authored the text, and created the figures. AO, GF: oversaw the text and assisted in the interpretation of the findings. TL: principal TKA surgeon and oversaw data collecting. MMP: oversaw the preparation of the report, helped interpret the findings and planned the study. Each author contributed to the development of the research and manuscript by offering constructive criticism.

Handling co-editors: Marianne Westberg and Robin Christensen

*Acta* thanks Lars Nordsletten for help with peer review of this manuscript.
